# How the Kinetic Balance Between Charge‐Transfer and Mass‐Transfer Influences Zinc Anode Stability: An Ultramicroelectrode Study

**DOI:** 10.1002/smtd.202401021

**Published:** 2024-11-20

**Authors:** Ashutosh Rana, Md. Arif Faisal, Kingshuk Roy, James H. Nguyen, Saptarshi Paul, Jeffrey E. Dick

**Affiliations:** ^1^ Department of Chemistry Purdue University West Lafayette IN 47907 USA; ^2^ Research Institute for Sustainable Energy Center for Research and Education in Science and Technology (TCG‐CREST) Salt Lake Kolkata 700091 India; ^3^ Elmore Family School of Electrical and Computer Engineering Purdue University West Lafayette IN 47907 USA

**Keywords:** aqueous zinc metal batteries, electrodeposition kinetics, fast‐scan voltammetry, mass transfer, ultramicroelectrodes

## Abstract

Aqueous zinc‐metal batteries (AZMBs) represent a promising frontier in battery technology, offering sustainable and safe alternatives to traditional non‐aqueous batteries. Despite their potential, understanding the kinetics of zinc electrodeposition—a critical factor in AZMB performance—remains underexplored. Utilizing voltammetry on ultramicroelectrodes, we investigate how scan rate influences key processes of nucleation and growth during Zn^2+^ electrodeposition. The findings highlight the efficacy of the Butler‐Volmer formulation in capturing electron‐transfer kinetics, contrasting with complex electron transfer kinetic models used for non‐aqueous battery chemistries. We clearly demonstrate that there is a strong dependence of scan rate on the measured value of kinetic parameters (exchange current). To accurately probe the charge transfer kinetics, it is essential to apply fast scan voltammetry to decouple the influence of mass transfer, ensuring that the measured current is independent of the scan rate. Furthermore, by studying a model electrolyte additive, the intricate balance between charge transfer and mass transfer dynamics is unveiled, and this information is crucial for enhancing the stability of zinc metal anodes. These insights pave the way for developing advanced electrolyte and current collector formulations, promising enhanced cyclability and sustainability in zinc metal batteries.

## Graphical abstract

This study examines scan rate effects on Zn²⁺ nucleation and growth during electrodeposition using voltammetry on ultramicroelectrodes. The Butler‐Volmer model accurately describes electron‐transfer kinetics, emphasizing the need for fast scan voltammetry to decouple mass transfer effects. The study shows the critical impact of balancing charge and mass transfer kinetics, essential for stabilizing zinc anodes.

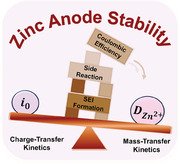

## 1 Introduction

The need for alternative battery chemistries, distinct from lithium‐ion batteries, arises from several factors driving advancements in energy storage technologies. Lithium‐ion batteries, while widely used and efficient, face challenges such as limited lithium resources, safety concerns, and environmental impacts associated with resource extraction and disposal. Aqueous zinc‐metal batteries (AZMBs) offer potential solutions to these challenges by leveraging abundant and sustainable materials, enhancing safety features, and reducing environmental footprint.^[^ ^1^, ^2^, ^3^ ^]^ The rapid development of AZMBs necessitates an in‐depth understanding of the factors influencing the kinetics of electron transfer (*Zn* ^2 +^ + 2*e* ^−^ →  *Zn* ^0^) occurring at the zinc electrode surface during the charging phase. To the best of our knowledge, this aspect has not been explored previously for AZMBs. This aspect has been explored in depth for non‐aqueous Li metal batteries, where the electrodeposition kinetics of Li (*Li* ^+^ + *e* ^−^ →  *Li* ^0^) was found to be convoluted with the transport of ions through the solid electrolyte interphase (SEI).^[^ ^4^, ^5^, ^6^, ^7^, ^8^ ^]^ Boyle et al. took significant strides in deciphering the electrodeposition kinetics for Li metal, emphasizing the importance of fast‐scan cyclic voltammetry (FSCV) for understanding the kinetics of electrodpeosition of Li.^[^ ^5^, ^9^ ^]^ Their work underscored the importance of FSCV for two primary reasons: it minimizes the influence of SEI on the measurement, and it positions the experiment under kinetic control at the lithium‐electrolyte interface, as opposed to mass transport control through the electrolyte or an SEI. The rapid advancement of AZMBs is currently plagued by issues such as dendrite formation during zinc electrodeposition, corrosion of the zinc metal, hydrogen evolution reaction, and other parasitic side reactions. To mitigate these drawbacks, several strategies are employed, such as the addition of additives, co‐solvents, current collector modification, separator modification, etc.^[^ ^10^, ^11^, ^12^, ^13^ ^]^ In all of these scenarios, an understanding of electrodeposition kinetics is essential both before and after perturbing the native system with the aforementioned modification strategies.

In general, achieving an ultra‐stable anode for AZMBs requires a uniform, dense, dendrite‐free morphology, which is closely tied to the nature of the SEI formed. The morphology of electrodeposited zinc primarily depends on two important factors: charge transfer kinetics (Zn^2^⁺ + 2e⁻ → Zn⁰) and mass transfer/ diffusion of Zn^2^⁺ ions to the electrode surface.^[^ ^5^, ^10^, ^14^ ^]^ In AZMB literature, more importance is given to the former, while the latter is often ignored. However, there is a strong interplay between these two factors that governs the overall stability of the zinc metal anode. Over the years, the community has crystallized on the understanding that a slower rate of electron transfer (lower values of exchange current) is associated with suppressed side reactions, uniform morphology, and a stable zinc metal anode.^[^ ^11^, ^15^, ^16^, ^17^, ^18^, ^19^ ^]^ The rate of electron transfer is often evaluated using Tafel analysis which simply translates as a high‐overpotential approximation of Butler‐Volmer formulation.^[^ ^20^ ^]^ However, the literature lacks clear guidelines and considerations for interpreting Tafel analysis results for AZMBs. Moreover, no work delves into the intricacies of the interplay between charge transfer and mass transfer's influence on the stability of the zinc metal anode. Therefore, in this work, we detail the various factors influencing the kinetics of electrodeposition for AZMBs using ultramicroelectrodes (UMEs) from an electroanalytical perspective. We provide a clear understanding, guidelines, and considerations on evaluating electron transfer kinetics and incorporating the role of mass transfer into the overall understanding. Furthermore, by studying a model electrolyte additive, we unveil the intricate balance between charge transfer and mass transfer dynamics, crucial for enhancing the stability of zinc metal anodes. Our proposed methodologies and ideas will pave the way for engineering advanced electrolyte and current collector formulations, ultimately aimed at achieving long‐term cyclability of zinc metal batteries.

## 2 Results and Discussion

Throughout this work, we employ voltammetric measurements using UMEs due to their low cell time constant (τ  =  *R_u_C_d_*, where *R_u_* is the uncompensated resistance and *C_d_* is the double‐layer capacitance), allowing us to make measurements in a shorter time domain. The value of τ sets the lower limit for the experimental timescale. Typically, millimeter‐sized electrodes (macroelectrodes) are limited to the millisecond time domain, while for microelectrodes, this experimental timescale drops to the microsecond range.^[^ ^20^ ^]^ The following section is divided into several sub‐sections. First, we detail the use of FSCV for understanding electrodeposition kinetics of zinc, followed by an exploration of nucleation and growth kinetics. The last section delves into the interplay of charge transfer and mass transfer by examining the influence of an organic electrolyte additive reported to slow down desolvation kinetics and enhance the overall stability of zinc metal anode.

### 2.1 Electrodeposition Kinetics of Zinc

**Figure 1:**
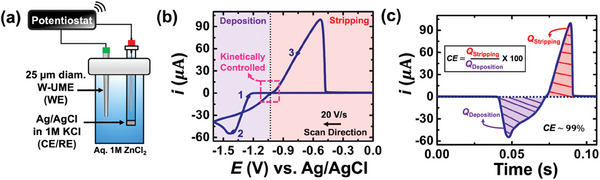
a) Schematic illustration of the experimental setup used for performing the cyclic voltammetry. A 25 µm diameter Tungsten ultramicroelectrode serves as the working electrode, while an Ag/AgCl electrode in 1 M KCl is used as the reference/counter electrode. b) Fast‐scan cyclic voltammogram recorded for the electrodeposition of zinc on a tungsten UME at a scan rate of 20 Vs^−1^. The current versus potential plot shown in (b) is converted to current versus time (c), allowing us to determine the coulombic efficiency (CE) for the electrodeposition of zinc.

The experimental setup for performing FSCV is depicted in **Figure** **1**a, utilizing a two‐electrode configuration. A 25 µm diameter Tungsten UME (W UME) serves as the working electrode, while an Ag/AgCl electrode in 1 m KCl is used as the reference/counter electrode. Due to the small size of the UME, the current passed through it is negligible, enabling experiments to be conducted using a two‐electrode setup. Generally, a three‐electrode setup is applied when the working electrode is large; the counter electrode completes the circuit, and the reference electrode, whose stable equilibrium is incredibly important for quantitation, is protected. Because of the size of UMEs, the amount of charge passed during voltammetry is small compared to a macroelectrode. Thus, the reference electrode can also be used to complete the circuit without deleterious effects to its important surface equilibrium. In this study, we chose to work with W and Cu UMEs because these electrode materials are commonly utilized for voltammetric measurements relevant to batteries. A cyclic voltammogram recorded for the electrodeposition of Zn^2+^ on W UME is shown in Figure 1b. The voltammetry was performed between 0 and −1.6 V at a scan rate of 20 Vs^−1^ (see arrow for the scan direction). The plot has been color‐coded with two different shaded areas: purple and red, indicating the potential range associated with deposition and stripping of zinc, respectively. An IUPAC convention is used, where cathodic current (*Zn* ^2 +^ → *Zn* ^0^, deposition) is negative, and anodic current (*Zn* ^0^ → *Zn* ^2 +^, stripping) is positive. On the forward sweep, the initial nucleation (point 1 in Figure 1b) of zinc on the W UME is observed at −1.2 V versus Ag/AgCl, followed by an increase in current leading to the presence of a peak (−1.35 V, point 2 in Figure 1b) that arises due to diffusion limitation. The peak will allows us to extract diffusion coefficient ($D_{Zn^{2+}}$) of Zn^2+^ in the electrolyte, as will be shown later in the work. The voltammetric sweep is then reversed at −1.6 V versus Ag/AgCl, leading to a nucleation loop, which is characteristic of the electrodeposition of metals.^[^ ^5^, ^20^, ^21^, ^22^ ^]^ Note that even after switching the direction of voltage sweeps, zinc deposition continues until a cross‐over is observed from the purple region to the red region. The crossover potential is observed at −1.02 V, which approximately equals the open circuit potential when measured using a zinc foil as the working electrode, Pt as the counter electrode, and Ag/AgCl as the reference electrode (see Figure S2, Supporting Information). This suggests that the crossover potential is equivalent to 0 V versus Zn^2+^/Zn^0^. This crossover potential will be used later for conversion of potentials measured with Ag/AgCl to Zn/Zn^2+^.

Upon sweeping to a more positive potential, we observe a positive surge of current (point 3) indicating the stripping of the electrodeposited zinc, followed by a stripping peak. The magnitude of current at the peak will be referred to as the peak current (*i_p_*), and its position on the x‐axis will be referred to as the peak potential (*E_p_*). The value of *i_p_* and *E_p_* highly depends on the scan rate of the voltammetric experiment (vide infra). The region delineated by the pink box in Figure 1b indicates the region of kinetic control, identified by the region where the current is under 10% of its peak current value. It has previously been shown that this region can be used to fit kinetic models with negligible error.^[^ ^20^, ^23^ ^]^ This region is distinguished by charge‐transfer kinetics as the rate‐limiting step, unaffected by mass transfer effects. This region can be utilized to understand the charge transfer kinetics of electrodeposition and calculate kinetic parameters such as the exchange current (*i*_0_). This can be achieved through several methods, such as using a linear‐low‐overpotential approximation of Butler‐Volmer equation, applying the Butler‐Volmer equation under no influence of mass transfer, or utilizing other complex models like the Marcus theory of electron transfer by incorporating an overpotential‐dependent transfer coefficient.^[^ ^20^ ^]^ It is important to note that the Butler‐Volmer equation is primarily designed for simple one‐electron elementary outer‐sphere reactions, whereas electrodeposition involves more complex, multi‐step inner‐sphere reactions. Despite the model's simplicity, it remains the gold standard and allows for a comparison amongst datasets. For the most part of this work, we opt for Tafel Analysis as it is the most commonly used technique to determine the kinetics of electrodeposition. The general framework for performing Tafel Analysis relevant to electrodeposition involves obtaining a cyclic voltammogram (current vs potential), identifying the kinetic regime, and plotting Log_10_(|current|) versus overpotential within that regime. The Tafel equations (Equations S1 and S2, Supporting Information) for anodic and cathodic reactions allow for the calculation of the exchange current (*i*_0_) from the intercept value of the linear regime. The values of *i*_0_ serve as a crucial metric for electrodeposition and suggest several properties of the system, such as the extent of side reactions, desolvation kinetics of solvated Zn^2+^ ions prior to electrodeposition, passivation, etc.^[^ ^10^, ^15^, ^24^, ^25^, ^26^, ^27^, ^28^ ^]^ It will be later shown that along with the values of exchange current, mass transfer of solvated zinc ions (i.e., $D_{Zn^{2+}}$) will be a crucial metric in determining the overall stability of zinc metal anode. A commonly overlooked metric in voltammetry for electrodeposition of metals is the charge associated with deposition and stripping. Figure 1c depicts the current versus time trace, derived from the cyclic voltammogram illustrated in Figure 1b. The x‐axis represents the time scale of the experiment. The total duration of the voltammogram displayed in Figure 1b was 0.11 s. The deposited charge was measured to be 1.23 µC, while the stripping charge was 1.22 µC, resulting in a coulombic efficiency (CE) of ≈99%. The deposited charge will indeed emerge as a vital metric, as its significance will become apparent later on.

**Figure 2:**
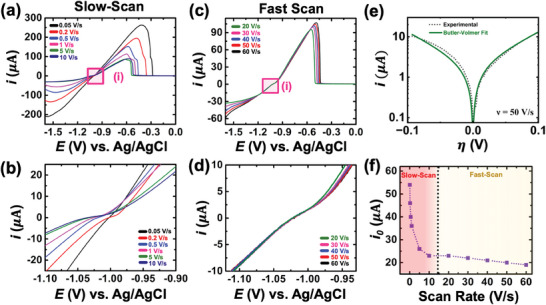
a) Backward sweep of the cyclic voltammogram recorded at scan rates of 50 mV s^−1^ to 10 V s^−1^. Inset (i) shows the kinetic regime of the voltammograms. A zoomed‐in image of the kinetic regime is shown in (b). c) Backward sweep of the cyclic voltammogram recorded at scan rates of 20 V s^−1^ to 60 V s^−1^. Inset (i) shows the kinetic regime of the voltammograms. A zoomed‐in image of the kinetic regime is shown in (d). e) Kinetic regime (10% peak current) showing the experimental and Butler‐Volmer fit in the backward sweep of voltammogram with scan rate 50 V s^−1^, allowing us to calculate the exchange current from the y‐intercept. f) Exchange current as a function of scan rate.

**Figure** **2**a depicts the backward sweep (−1.6 to 0 V) of the cyclic voltammograms at varying scan rates. It's evident that there is a notable change in the observed peak current and peak potentials as the scan rates are varied. Specifically, the peak current dramatically decreases with an increase in the scan rate from 50 mV s^−1^ to 10 V s^−1^. We refer to these particular scan rates as belonging to the “slow scan rate” regime. This observation might seem counterintuitive when considering the voltammetry of reversible outer sphere molecules, where the peak current scales with the scan rate according to the Randles‐Sevcik equation (*i_p_* ∝ν^1/2^).^[^ ^20^ ^]^ However, it's crucial to remember that these two systems are fundamentally different from each other as in the case of electrodeposition, as the scan rate is increased (i.e., the time scale of measurement is decreased), we deposit a lesser amount of material, resulting in a lower value of stripping peak current. The kinetic regime is depicted by the shaded pink region marked by (i) in Figure 2a, with a magnified view provided in Figure 2b. It has been shown in literature for electrodeposition of Li that the low scan rate regime represents a Nernstian or mass‐transport limited electrodeposition process, where the scan rate alters the current at all points, with a fixed peak potential value at all scan rates.^[^ ^4^, ^5^, ^6^, ^7^, ^8^, ^9^, ^14^ ^]^ However, for the case shown in this work, it does not agree, as the peak potential shifts with the scan rate and peak currents decrease with the scan rate as well.

Overall, two clear observations can be made in the low‐scan regime: first, the crossover potential is not stable, and second, the nature of these curves (slope) varies significantly, with the slope decreasing as the scan rate increases. This indicates that the slow scan‐rate kinetic regimes are perturbed by mass‐transfer limitations.

**Figure 3:**
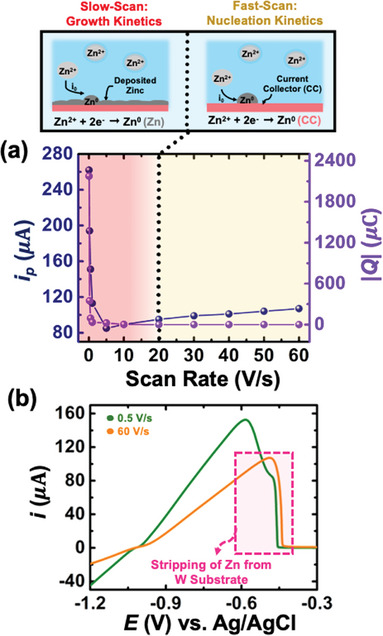
a) Double‐axis plot showing the variation of peak current (*i_p_*) and deposited charge (*Q_dep_*) as a function of scan rate. b) Stripping peaks for backward sweeps for 0.5 Vs^−1^ and 30 Vs^−1^, showing the presence of a shoulder peak at low scan rates, and the position of the peak at high scan rates aligns with the shoulder peak.

Figure 2c,d shows the backward sweep of the voltammograms recorded at scan rates higher than 10 Vs^−1^. Increasing the scan rate beyond 10 Vs^−1^ reveals an interesting observation: the peak current increases with increasing scan rate. We refer to these particular scan rates as belonging to the “fast scan rate” regime. This is surprising as there is an inversion in the trend of the peak current value. The results at fast scan rates exhibit a characteristic kinetically irreversible/quasi‐reversible nature: only the peak current scales with the scan rate and the peak potential shifts with scan rate, while the current in the kinetic regime remains independent of the scan rate. This indicates that the fast scan‐rate kinetic regimes are not perturbed by mass‐transfer limitations and truly report on the electron‐transfer kinetics. Moreover, the kinetic regime exhibits altogether different characteristics, with a stable crossover potential as well as same slopes for all the curves. The results depicted for fast‐scan rates, where the peak current increases with scan rate, and similar kinetic regimes for all the scan rates (20–60 Vs^−1^), are consistent with measurements reported in literature for the electrodeposition of Li metal on W UME.^[^ ^5^ ^]^ Additionally, in the slow‐scan rate experiments depicted in Figure 2a, the voltammetric traces exhibit the presence of a shoulder peak alongside the stripping peak. It's important to note that such behavior is not observed in the voltammograms at high‐scan rates (see Figure 3b). The results suggest that the larger stripping peak corresponds to zinc being stripped from the surface of the electrodeposited zinc, while the shoulder peak indicates zinc stripping from the bare tungsten substrate. This implies that at low scan rates, the stripping process is dominated by growth kinetics rather than nucleation kinetics. To validate this, we conducted an independent experiment using a 125 µm diameter W working electrode with in‐situ monitoring of the morphology evolution at the electrode surface (see Movie S1, Supporting Information). It's evident that the second stripping peak closely aligns with the abrupt stripping of deposited zinc, revealing the fresh tungsten electrode surface, indicating that the second peak corresponds to the stripping of Zn from the bare electrode surface. This aspect will be discussed in more detail in the subsequent section.

The kinetic regime is often used to perform Tafel Analysis to calculate the exchange current (*i*_0_). Figure 2e shows a representative Tafel plot (dotted green curve; note that the y‐axis represents a Log scale and x‐axis represents the overpotential (η) vs Zn/Zn^2+^) using the kinetic regime for the polarization curve of scan rate 50 Vs^−1^. Along with the intercept value, the slope of the linear regime allows us to gain insights into the mechanism of the reaction.^[^ ^20^ ^]^ It's worth noting that the Tafel plot exhibits symmetry for the cathodic and anodic reactions, indicating similar values of exchange current for plating and stripping processes. Moreover, in light of research on non‐aqueous battery chemistries where it was found that Tafel analysis/Butler‐Volmer Model cannot accurately capture the electron‐transfer kinetics due to the need for a potential‐dependent transfer coefficient to account for solvation/reorganization energetics (Marcus theories of electron transfer).^[^ ^5^, ^29^, ^30^, ^31^ ^]^ Here, we found that the Butler‐Volmer model could accurately describe the electron‐transfer kinetics for AZMBs when the *i* versus η experimental curve was fit in the region associated with ± 10% values of the peak current. The solid green curve on the dotted curve in Figure 2e shows a non‐linear least square fit of the current‐overpotential equation (*i* = *i*_0_ (*e* ^(1−α)*f*η^ − *e* ^−α*f*η^), where *i*_0_ is the exchange current, η is the overpotential, α is the transfer coefficient (assumed to be 0.5), and *f* is *F*/*RT*, with *F* being the Faraday constant, *R* the universal gas constant, and *T* the temperature) with *i*_0_ as the fitting parameter.^[^ ^20^ ^]^ Based on the fit shown in Figure 2e, it is clear that the Butler‐Volmer model can accurately capture the electron‐transfer kinetics in AZMBs. A true linear regime without the presence of any curvature is observed at high overpotentials from the equilibrium overpotential, demonstrating that a fixed value of the transfer coefficient (0.5) accurately describes the electron transfer without the need to introduce a potential dependence for the transfer coefficient. Figure 2f presents the *i*_0_ values as a function of scan rate determined using Tafel analysis in a potential window of ±300 mV overpotential range (see Figure S3, Supporting Information for Tafel plots at different scan rates). At a scan rate of 50 mVs^−1^, the *i*_0_ value is 53 µA. With increasing scan rates, the *i*_0_ value decreases, eventually plateauing in the fast‐scan regime (20–60 Vs^−1^) with minimal change compared to the slow scan rates. In the literature, Tafel analysis is typically performed using linear sweep polarization curves with scan rates in the range of tens of mVs^−1^, but these scan rates are not standardized. The results presented earlier are concerning as there is a significant influence of scan rate on the measured values of *i*_0_, raising a very important question: What is the correct scan rate at which one should perform Tafel Analysis, and more importantly, why is such a trend observed for the values of peak current (*i_p_*) and exchange current (*i*_0_)? This question will be addressed in detail in the following sections.

### 2.2 Nucleation and Growth Kinetics of Electrodeposited Zinc

In order to understand the trend of the *i*_0_ values as a function of scan rate, we performed charge quantification for all the scan rates. The current versus time sweep for all the voltammograms presented in Figure 2 is shown in Figure S4 (Supporting Information), enabling us to determine the deposited charge for all the scan rates (Table S1, Supporting Information). **Figure** **3**a shows a double y‐axis plot with peak current (*i_p_*) and deposited charge (*Q_dep_*) as a function of scan rate. As expected, in the slow scan regime (<20 Vs^−1^), the deposited charge drops as the peak current drops (see Table S1, Supporting Information for exact values). However, in the fast scan regime (>20 Vs^−1^), the values of *Q_dep_* approach a plateau, where the decrease in charge is marginal. Therefore, we hypothesize that the observed trend of *i*_0_ values (Figure 2f) arise due to depositing a very similar amount of zinc in the fast scan regime, whereas the deposited charge in the slow scan regime varies significantly. It is important to realize that the current in the kinetic regime is directly proportional to the true electroactive area of the electrode (and not the geometric area), which significantly changes (increases) as a function of the amount of electrodeposited zinc. This implies that a similar amount of deposit on the electrode (*Q_dep_*) will exhibit a similar characteristic of the curves in the kinetic regime. In the literature, it is a common practice to use the geometric area of the electrode to obtain the exchange current densities. Based on our results, it is clear that it is not optimal to do so, as the electroactive area is constantly evolving and is a strong function of the amount of mass deposited on the electrode (or the scan rate/the time scale of the experiment).

As discussed earlier, the slow scan rate experiments (50 mVs^−1^ − 20 Vs^−1^) reveal the presence of two peaks, indicating two regions for stripping: first from the surface of electrodeposited zinc, followed by stripping from the bare electrode substrate. This observation turns out to be crucial as the position of the stripping peak at 60 Vs^−1^ (solid orange curve) aligns with the shoulder peak (solid green curve) observed in the lower scan rate experiments, as illustrated in Figure 3b, indicating that at high‐scan rates, we are primarily stripping from the bare electrode surface. This suggests minimal evolution of surface area at fast‐scan rates, resulting in similar characteristic curves in the kinetic regime and hence similar *i*_0_ values. Overall, these findings illustrate that the measured *i*_0_ values obtained at fast scan rates represent the nucleation kinetics of zinc on the bare tungsten substrate, while lower scan rates reflect the growth kinetics of zinc on pre‐deposited zinc on the tungsten substrate (see schematic on top of Figure 3a). In the literature on non‐aqueous battery chemistries, it is evident that slow scan rates can lead to side reactions, resulting in the formation of a SEI, which convolutes voltammetric measurements and yields much higher exchange current values compared to fast scan voltammetry.^[^ ^4^, ^5^, ^9^ ^]^ We rule out this possibility for AZMBs by evaluating the coulombic efficiency of the voltammograms at all scan rates. If side product formation were significant at slow scan rates, the coulombic efficiency would be lower at these rates. However, this is not the case (see Table S1, Supporting Information). In fact, it was observed that slow scan rate experiments have higher efficiency compared to fast scan voltammetry experiments.

Additionally, the consistent Tafel slopes (*m*) values across all scan rates suggest a similar mechanism for zinc electrodeposition (Table S1, Supporting Information). The results presented this far highlight the impact of deposited charge on the calculated exchange current values, especially in the slow scan rate regime. This underscores two important considerations: first, the non‐standardized nature of scan rates used to obtain polarization curves and Tafel plots in literature, and second, the necessity of fast scan voltammetry for accurately probing the charge‐transfer kinetics of zinc electrodeposition. These aspects are crucial, and in the following sections, we present two scenarios to further support this.

We conducted an independent experiment using two different 25 µm W UMEs (UME 1 and UME 2), where a similar amount of zinc was deposited on both electrodes. Figure S5a (Supporting Information) shows a linear sweep voltammogram depicting the deposition of zinc on UME 1 and UME 2. The same scan rate of 1 Vs^−1^ was chosen for both electrodes, resulting in the deposition of a similar amount of zinc on both the W‐UMEs, equaling ≈6.3 µC. We performed another linear sweep voltammetry from the crossover potential to a positive potential to strip the deposited zinc from both UMEs. The voltammetry was conducted at two different scan rates: UME 1 was stripped at 1 Vs^−1^, while UME 2 was stripped at 30 Vs^−1^. It's worth noting that these two scan rates belonged to the two different regimes observed in Figure 2, that is, low scan rate and high scan rate. In the results shown in Figure 2, the exchange current calculated for 1 and 30 V s^−1^ varied significantly, with *i*_0_ being 36 µA for 1 Vs^−1^ and 22 µA for 30 Vs^−1^. However, upon depositing the same amounts as shown in inset (i) of Figure S4b (Supporting Information), similar values for exchange current density were observed (11 µA for UME1 and 12 µA for UME2, respectively). Along with the influence of scan rate on the amount of deposited charge, the voltage window in which the cyclic voltammetry is performed also influences the deposited charge. Figure S5c (Supporting Information) shows cyclic voltammograms recorded at a scan rate of 1 Vs^−1^ with variations in the potential at which the voltammetric sweep is reversed (−2, −1.8, and −1.6 V vs Ag/AgCl). Tafel Analysis was performed to determine the exchange current values (47, 34, and 28 µA for −1.6, −1.8, and −2 V as the points at which the potential is reversed) for the curves show significant variations. These variations can be explained by the amount of deposited charge in each of the cases, as shown in Figure S5e (Supporting Information), where the deposited charge was determined as 200 µC, 87 µC, and 34 µC for −2, −1.8, and −1.6 V as the points at which the potential is reversed. Overall, these results provide strong evidence for the dependence of the values of *i*_0_ on the amount of deposited zinc, suggesting that the differences in the deposition amount of zinc across various scan rates in the low regime and high regime (Figure 2) could be a potential reason for the observed trend in the values of exchange current.

Based on the findings presented so far, it is evident that fast‐scan voltammetry would yield more accurate estimates of kinetic parameters (*i*_0_) compared to low‐scan rate voltammetry. This conclusion rests on two primary reasons: First, fast‐scan voltammetry can explore kinetic regimes in voltammograms that are not influenced by mass‐transfer effects. Second, the evolution of the electrode's surface area is minimal under fast scan conditions, thereby reducing the impact of changes in the electrode's electroactive surface area on the observed current in voltammetry. However, accessing high scan rates typically requires the use of microelectrodes rather than commonly used macroelectrodes (100 µm‐ mm sized electrodes) because the non‐faradaic charging current increases linearly with scan rate, and the faradaic response increases with the square root of scan rate. Despite this, conducting measurements on macroelectrodes at low to moderate scan rates can still provide meaningful comparisons of the growth kinetics and yield relative trends in the system, provided that caution is exercised regarding the amount of deposited charge. For instance, when demonstrating the modulation of desolvation kinetics using an electrolyte additive based on Tafel Analysis to understand the sluggish nature of electron transfer, it is crucial to be cognizant of the amount of zinc deposited in both systems before performing stripping/deposition to obtain a Tafel plot. This precaution is necessary because a lower amount of deposited charge in the presence of the additive can lead to a lower measured exchange current, not necessarily due to the sluggish nature of electron‐transfer kinetics, but rather because of a lower electroactive surface area. Consequently, comparisons between the two systems may no longer be meaningful.

### 2.3 Interplay Between Charge‐Transfer and Mass‐Transfer Kinetics

At this point, we can pose a crucial question: what determines the transition from the slow‐scan rate regime, where the kinetic regime is affected by mass transport limitations, to the fast‐scan rate regime, where the kinetic regime is solely governed by electron‐transfer kinetics?

**Figure 4:**
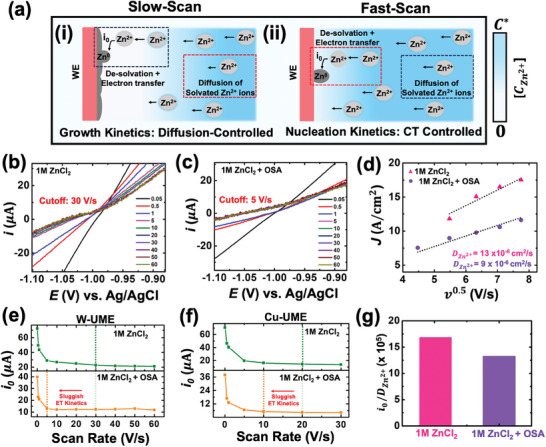
a) Schematic representation of the rate limiting steps during electrodeposition at slow scan rates and fast scan rates. b) Backward sweep of the cyclic voltammogram recorded at scan rates of 50 mVs^−1^ to 60 Vs^−1^ in 1 M ZnCl_2_. c) Backward sweep of the cyclic voltammogram recorded at scan rates of 50 mVs^−1^ to 60 Vs^−1^ in 1 M ZnCl_2_ + OSA. d) Diffusion coefficient ($D_{Zn^{2+}}$) of Zn^2+^ in presence and absence of OSA in 1 M ZnCl_2_. Comparison of exchange current (*i*_0_) in presence and absence of OSA in 1 M ZnCl_2_ on a tungsten (e) and copper (f) UME. g) Ratio of exchange current (*i*_0_) and diffusion coefficient ($D_{Zn^{2+}}$) in presence and absence of OSA in 1 M ZnCl_2_.

To understand this, let's step back and examine the sequential steps involved in the electrodeposition process. When a bias (voltage/ current) is applied for electrodpeosition of zinc, Zn^2+^ diffuse toward the electrode surface. Subsequently, these ions undergo desolvation, where the solvated water molecules surrounding them are removed. Following desolvation, the ions adsorb onto the electrode surface and undergo actual electron transfer.^[^ ^10^, ^32^ ^]^ The electron‐transfer step occurs very rapidly and is often convoluted with the desolvation process. Collectively, we refer to the combination of these two processes as charge transfer. Considering the series of sequential processes involved in electrodeposition, cyclic voltammograms provide insights into the rate‐limiting step at a given scan rate. At slow scan rates, the experimental time scale strongly influences the kinetic regime and exchange current values, indicating a diffusion‐controlled process. It is also well‐known that the growth phase during electrodeposition is often diffusion‐limited.^[^ ^5^ ^]^ It is worth noting that diffusion‐limited growth, which occurs as a result of concentration polarization, triggers the formation of dendrites during electrodeposition.^[^ ^14^, ^33^ ^]^ This is detrimental to the long‐term cyclability of zinc metal anodes. Conversely, at fast scan rates, the experimental time scale (scan rate) has no significant influence on the kinetic regime and exchange current values, suggesting a charge‐transfer‐controlled process. This overall process is summarized in schematic form in **Figure** **4**a. This analysis clearly explains the reason for the transition between slow‐scan and fast‐scan regimes: there is a shift in the rate‐limiting step from mass transfer to charge transfer.

To validate the proposed hypothesis, we demonstrate how a commonly reported organo‐sulfur additive (OSA), which promotes sluggish desolvation kinetics, and maximizes the stability of zinc anodes, affect the voltammograms/kinetic parameters.^[^ ^10^, ^15^, ^34^, ^35^, ^36^ ^]^ Organo‐sulfur electrolyte additives (OSAs) are known to alter the solvation matrix of Zn^2+^ ions by preferentially replacing water molecules.^[^ ^10^, ^15^ ^]^ This occurs because OSAs have a higher donor number compared to water molecules, allowing them to bind more effectively to Zn^2+^ ions. Figure 4b,c shows the kinetic regimes from the backward sweep of the cyclic voltammogram recorded at scan rates of 50 mVs^−1^ to 60 Vs^−1^ in presence and absence of OSA in 1 M ZnCl_2_. Clearly, in the presence of OSA the transition from slow to fast scan regimes occurs at 5 Vs^−1^ versus 30 Vs^−1^ in absence of OSA. We refer to these transitions from slow to fast scan rates based on the nature of the voltammograms in the kinetic regime. In the slow scan regime, curves vary significantly due to the influence of mass transfer, whereas in the fast scan regime, they remain stable as mass‐transfer effects are negligible. This occurs due to sluggish charge transfer kinetics in the presence of OSA, facilitating the transition from a mass‐transfer‐controlled regime to a charge‐transfer‐controlled regime at comparatively lower scan rates. Understanding this transition in scan rates can significantly enhance the comprehension of the role of additives in electrolytes from an electroanalytical perspective, which is currently absent in the literature.

The diffusion coefficient in the presence and absence of OSA in 1 M ZnCl_2_ was determined using the peak observed during the forward sweep of the cyclic voltammograms (refer back to point 2 in Figure 1b). The equation of peak current as a function of scan rate for irreversible redox reaction,

(1) $$j_{p}=\left(2.99\times 10^{5}\right)n^{\frac{3}{2}}\;C_{Zn^{2+}}\;\alpha^{\frac{1}{2}}\;D_{Zn^{2+}}^{\frac{1}{2}}\;v^{\frac{1}{2}}$$

was used to determine the diffusion coefficient using the slope of the fit between *j_p_* and ν^0.5^, as shown in Figure 4d.^[^ ^5^, ^9^, ^20^ ^]^ Where, *j_p_* is the peak current density (peak current divided by geometric area), *n* is the number of electrons transferred, $C_{Zn^{2+}}$ is the concentration of zinc ions, α is the transfer coefficient, $D_{Zn^{2+}}$ is the diffusion coefficient of Zn^2+^ and ν is the scan rate. It is important to address that while the Randles‐Sevcik equation is commonly used in the literature to determine diffusion coefficient values by analyzing the slopes of plots of peak current versus the square root of the scan rate, we utilize equations related to irreversible kinetics in this work. The Randles‐Sevcik equation is typically applied to reversible outer‐sphere redox kinetics, where the peak potentials does shift as a function of the scan rate. However, our data on zinc electrodeposition show that the peak potential shifts with the scan rate, which is indicative of irreversible kinetics. This shift demonstrates that the Randles‐Sevcik equation, which is not applicable to irreversible processes, is not suitable for our analysis. The values of $D_{Zn^{2+}}$ were found to be slightly higher in the absence of OSA in ZnCl_2_. At a glance this might appear non‐ideal for the stability of the zinc anode, as a lower diffusion coefficient would lead to higher concentration polarization, which is known to trigger the formation of dendrites during electrodeposition. However, we must not ignore the charge transfer kinetics, which is discussed in the next paragraph.

Using the kinetic regimes in the presence and absence of OSA, exchange current values were derived for electrodeposition on tungsten UMEs, as shown in Figure 4e. The exchange current in the absence of OSA​ was calculated in the fast scan regime (>30 Vs^−1^) and was found to be 21 µA. In the presence of OSA, the exchange current was calculated in the fast scan regime (>5 Vs^−1^) and calculated to be 12 µA. These lower values in the presence of OSA are expected due to sluggish desolvation kinetics and suppressed side reactions, which lead to the formation of a robust, conductive SEI. To investigate the influence of the electrode substrate, the 25 µm tungsten UME was replaced by a 25 µm copper UME, and similar analysis was performed as shown in Figure 4e. A similar trend was observed, with a lower exchange current in the presence of OSA in ZnCl_2_. However, the cut‐off scan rates for the transition from slow to fast regimes were different. This is not surprising, as the fast scan voltammetry results report on nucleation kinetics, which are substrate‐dependent.^[^ ^4^, ^11^, ^37^ ^]^ At this point, we have two competing findings: a lower value of $D_{Zn^{2+}}$ in the presence of OSA, which is undesirable, and a lower value of *i*_0_ in the presence of OSA, which is desirable. However, if we carefully examine the values of the exchange current, it is almost halved in the presence of OSA, whereas the change in diffusion coefficient absence and presence of OSA is marginal. This suggests that the overall process is dominated by charge transfer in the presence of OSA in ZnCl_2_. This dominance of charge transfer is likely the reason for the enhanced stability of zinc metal anodes in the presence of OSA (see bar graph in Figure 4g). It is important to note that we do not claim that j_0_/D is a “universal” parameter in governing the stability of the zinc metal anode, considering the various factors that contribute to overall performance. However, this parameter and the analysis detailed in this work may provide researchers with insights from a fundamental perspective, potentially aiding in the interpretation of results. Overall, using a model additive, this analysis allows us to understand the interplay between mass‐transfer and charge‐transfer and its overall influence on the stability of the zinc anode. The proposed methodologies are expected to advance our understanding of electrodeposition kinetics in advanced electrolytes and current collector formulations, with the ultimate goal of achieving long‐term cyclability of zinc metal batteries.

**Figure 5:**
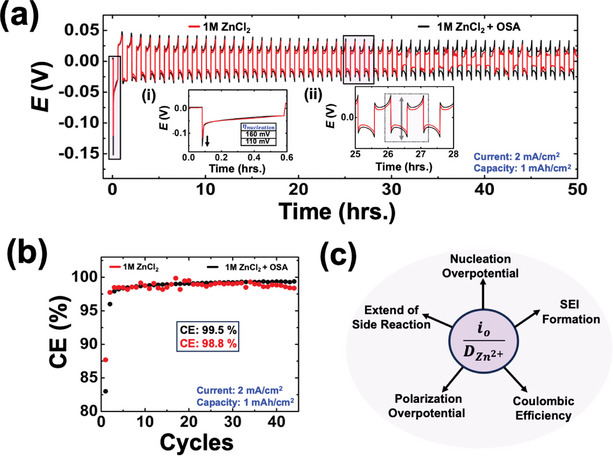
a) Zn|Zn symmetric cell cycling at a current density of 2 mA cm^−^^2^ and a capacity of 1 mAh cm^−^^2^ in 1 M ZnCl₂, comparing results with and without OSA. Inset (i) shows the nucleation overpotential of the first cycle and inset (ii) shows the overall voltage polarization. b) Coulombic efficiency of Cu|Zn asymmetric cell cycling current density of 2 mA cm^−^^2^ and a capacity of 1 mAh cm^−^^2^ in 1 M ZnCl₂ with a voltage cutoff of 0.8 V in the presence and absence of OSA in 1 M ZnCl_2_. c) Schematic showing the coin cell metrics influenced by the interplay of charge transfer and mass transfer during electrodeposition.

Now, we will examine how the interplay between mass transfer and charge transfer kinetics influences the commonly reported metrics for the stability of zinc metal anodes in symmetric (Zn|Zn) and asymmetric coin cells (Zn|Cu). It's important to note that the objective here is not to evaluate the overall enhanced stability of the zinc metal anode in the presence of OSA, as this has already been well‐established in the literature.^[^ ^10^, ^15^, ^34^, ^35^, ^36^ ^]^ **Figure** **5**a shows symmetric cell cycling at a current density of 2 mA cm^−^^2^ and a capacity of 1 mAh cm^−^^2^ in 1 M ZnCl₂, comparing results with and without OSA. Clearly, in the presence of OSA, the nucleation overpotential is higher (160 mV vs 110 mV in the absence of OSA, inset (i) of Figure 5a), and overall voltage polarization is also higher (inset (ii) of Figure 5a) in the presence of OSA. This outcome is expected due to the sluggish electrodeposition kinetics (lower exchange current values and similar diffusion coefficients) associated with OSA. A higher nucleation overpotential in the presence of OSA is advantageous as it reduces the critical radius of deposited zinc nuclei, leading to higher areal density and more homogeneous, non‐dendritic deposition.

Figure 5b illustrates the coulombic efficiency (CE) of asymmetric Zn|Cu cells cycling at a current density of 2 mA cm^−^^2^ and a capacity of 1 mAh cm^−^^2^ in 1 M ZnCl₂, comparing results with and without OSA over 45 cycles. It was observed that CE values exhibited significant fluctuations, averaging 98.8% in the absence of OSA. In contrast, with OSA, CE values were notably stable, averaging 99.5%. Furthermore, CE values stabilized within fewer than 10 cycles in the presence of OSA, whereas no stabilization in CE values was observed in its absence. These results directly translate from the enhanced stability, suppressed side reactions, and uniform SEI formation of the zinc metal anode in the presence of OSA, as discussed earlier. The combined effect on these coin cell metrics is summarized schematically in Figure 5c. The scheme illustrates that kinetic parameters strongly influence factors such as the nucleation overpotential, SEI formation, extent of side reactions, polarization overpotential, and overall long‐term stability/coulombic efficiencies of both symmetric and asymmetric cells. The findings presented in this study, employing UME from an electroanalytical standpoint, provide insights into the factors influencing electrodeposition kinetics. Notably, the application of fast scan voltammetry proves crucial for accurately probing electron transfer kinetics in AZMBs. The proposed methodologies and ideas will catalyze the development and understanding of cutting‐edge electrolyte and current collector formulations, aiming squarely at achieving enduring cyclability for zinc metal batteries. It is important to note that the current study utilizes tungsten and copper microelectrodes, which are not the commonly used anode materials in zinc metal batteries. As a result, these materials may not accurately reflect the true zinc electrodeposition kinetics on a bare zinc anode substrate. However, the methodology detailed in this work is directly relevant for studying electrodeposition on current collectors and evaluating asymmetric cell performance. This approach allows for meaningful comparisons within the system, enabling the analysis of relative kinetics while designing next‐generation batteries that incorporate electrolyte additives and other electrolyte formulations.

## 3 Conclusion

The rapid advancement of AZMBs necessitates a comprehensive understanding of zinc electrodeposition kinetics, a relatively unexplored area in current literature. Utilizing voltammetry on ultramicroelectrodes, this study investigates the influence of scan rate and deposited charge on the exchange current (*i*_0_), offering critical insights into nucleation and growth kinetics during Zn^2+^ electrodeposition. We demonstrate the efficacy of the Butler‐Volmer formulation in accurately describing electron‐transfer kinetics, contrasting with models used in non‐aqueous battery chemistries. The accurate use of the presented equations and best practices for analysis in battery research will be a topic of a future investigaion from our laboratory. Moreover, employing a model electrolyte additive, we elucidate the intricate interplay between charge transfer (electron transfer + desolvation kinetics) and mass transfer (Zn^2+^ ion diffusion), crucial for stabilizing zinc metal anodes. Our methodologies and findings pave the way for developing advanced electrolyte and current collector formulations, aimed at enhancing the long‐term cyclability and performance of AZMBs.

## Conflict of Interest

The authors declare no conflict of interest.

## Author Contributions

A.R., M.A.F., and K.R. contributed equally to this work. The concept of the work was conceived by A.R. All the experiments were performed by A.R., M.A.F., K.R., and J.H.N., J.E.D. supervised all aspects of the work. All authors have agreed to the final version of the manuscript.

## Supporting information



Supporting Information

Supplemental Movie 1

## Data Availability

The data that support the findings of this study are available from the corresponding author upon reasonable request.
