# Supplementary material for: How the Kinetic Balance Between Charge‐Transfer and Mass‐Transfer Influences Zinc Anode Stability: An Ultramicroelectrode Study
Source: Small Methods. 2024 Nov 20;9(3):2401021. doi: 10.1002/smtd.202401021 (PMC11926490; doi:10.1002/smtd.202401021)
Supplement: Supplementary file 1 — Supporting Information [file SMTD-9-2401021-s001.pdf]

small methods

Supporting Information

for *Small Methods*, DOI 10.1002/smtd.202401021

How the Kinetic Balance Between Charge-Transfer and Mass-Transfer Influences Zinc Anode Stability: An Ultramicroelectrode Study

*Ashutosh Rana, Md. Arif Faisal, Kingshuk Roy, James H. Nguyen, Saptarshi Paul and Jeffrey E. Dick**

Supplementary Information

Unveiling the Factors Influencing the Stability of Zinc Metal Anode: The Interplay of Charge-Transfer and Mass Transfer Revealed through Ultramicroelectrodes

Ashutosh Rana[a+], Md. Arif Faisal[a+], Kingshuk Roy[a+], James H. Nguyen[a], Saptarshi Paul[a] and Jeffrey E. Dick[a,b*]

[a] Department of Chemistry, Purdue University, West Lafayette, IN, 47907, USA

[b] Elmore Family School of Electrical and Computer Engineering, Purdue University, West Lafayette, IN, 47907, USA

Corresponding Author:

Jeffrey E. Dick (jdick@purdue.edu)

[+]Equal contribution

Experimental Section

1. Materials

Zinc Chloride ($ZnCl_2$, reagent grade), Hexaammineruthenium (III) chloride, Potassium Chloride (KCl, reagent grade) and Dimethyl sulfoxide (DMSO, reagent grade) were bought from Sigma Aldrich. Zinc foil (99.99%) was procured from MTI Supplies. 25 μm Tungsten and Copper metal wires were bought from Good Fellow. All the chemicals were used without further purification. A Milli-Q ultrapure water production system was utilized to obtain the deionized (DI) water needed to prepare all the aqueous electrolytes.

2. Preparation of the Electrolyte and Ultramicroelectrode

The electrolytes were prepared by dissolving 1 mol/L (M) $ZnCl_2$ in DI water at room temperature (25 °C). The tungsten ultramicroelectrodes were made by following five steps. Firstly, one side of a capillary (100 mm diameter) was sealed with a propane torch. Next, the tungsten wire was threaded into the capillary and tapped to the sealed bottom. Then, the capillary with the wire was sealed by a heated coil while the vacuum pump was connected to the capillary to avoid any bubble formation. After that, a connection was made to the sealed tungsten wire with an electrical wire. Finally, the sealed bottom of the capillary was polished by sandpaper to expose the tungsten tip and then it was smoothed by further polishing on polishing pads with alumina and DI water.

3. Method of Testing the W Ultramicroelectrode

Employing a two-electrode setup instead of the conventionally used three-electrode setup is not problematic when working with a UME as the working electrode. The current passed through the system for measurements is typically very small, thereby allowing the potential drop at the working electrode to be referenced against a stable potential provided by the Ag/AgCl in 1 M KCl reference/counter electrode. All the electrochemical measurements were conducted using 1 M $ZnCl_2$ as the electrolyte. Prior to conducting any zinc electrodeposition on the W UME, a cyclic voltammogram was recorded using a well-behaved outer-sphere redox molecule (Hexaammineruthenium (III) chloride, RHT) to ensure the observation of a steady-state current, as is typical for a UME. **Fig. S1** illustrates a sigmoid-shaped cyclic voltammogram obtained using the same experimental setup as described earlier, with 5 mM RHT as the electrolyte. The presence of low capacitance and steady-state currents confirms the accurate fabrication of the UME.

4. Electrochemical Measurements

A CHI6284E electrochemical workstation was used for all electrochemical measurements. As mentioned in the main publication, a two-electrode setup was used for CV measurements. An optical microscope (OM, Olympus) coupled to a CHI potentiostat in a three-electrode arrangement was used to conduct in situ optical microscopy coupled electrochemical experiments. Zn deposition and stripping were recorded using a specially built 125 μm W electrode and an optical cell. The reference electrode was Ag/AgCl, and the counter electrode was Pt.

5. Equations for Tafel analysis[1]

Cathodic Reaction: $Log|i| = Log(i_0) - \left(\frac{\alpha F}{2.3RT}\right)\eta$ **[S1]**

$$\textbf{Anodic Reaction: } Log|i| = Log(i_0) + \left(\frac{(1-\alpha)F}{2.3RT}\right)\eta. \qquad \textbf{[S2]}$$

Where i is current, η is overpotential, i_0 is exchange current, F is Faraday's constant, R is universal gas constant, α is the transfer coefficient.

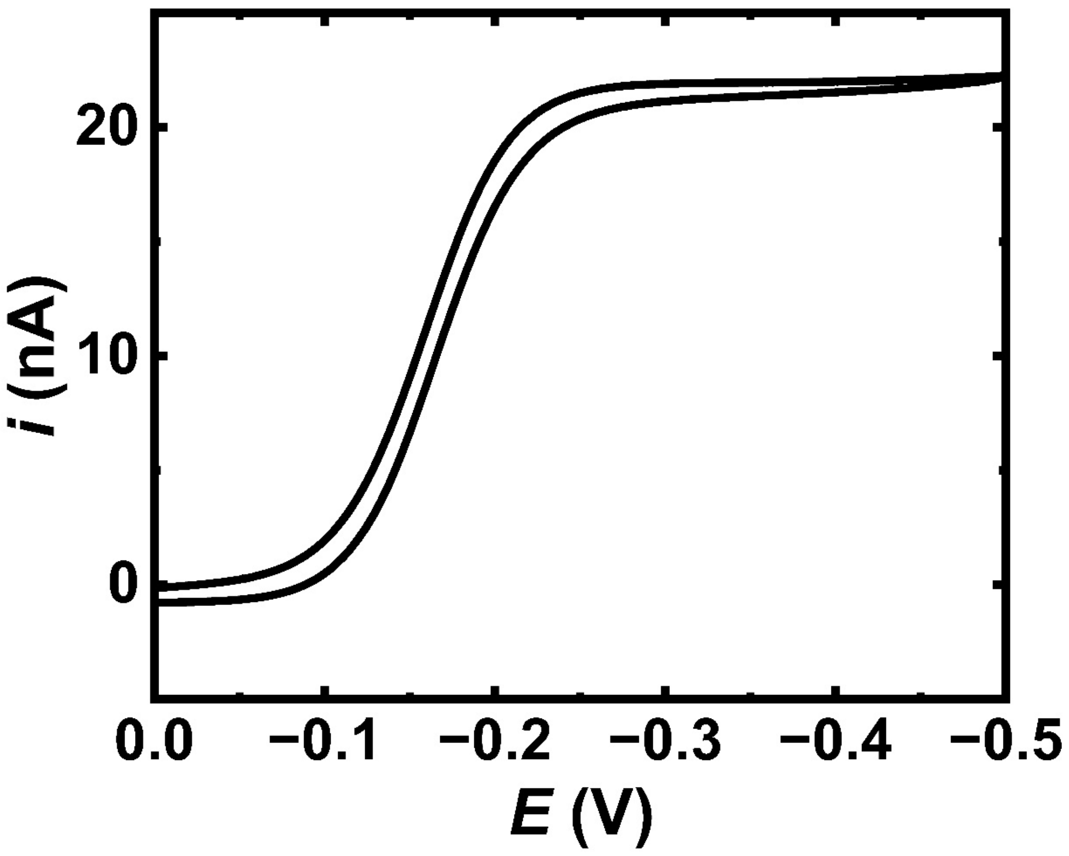

Fig S1. Cyclic voltammogram of a W UME in 5 mM Hexaammineruthenium (III) chloride in a two-electrode setup, where the fabricated W-UME served as the working electrode, and Ag/AgCl in 1 M KCl as the counter/ reference electrode.

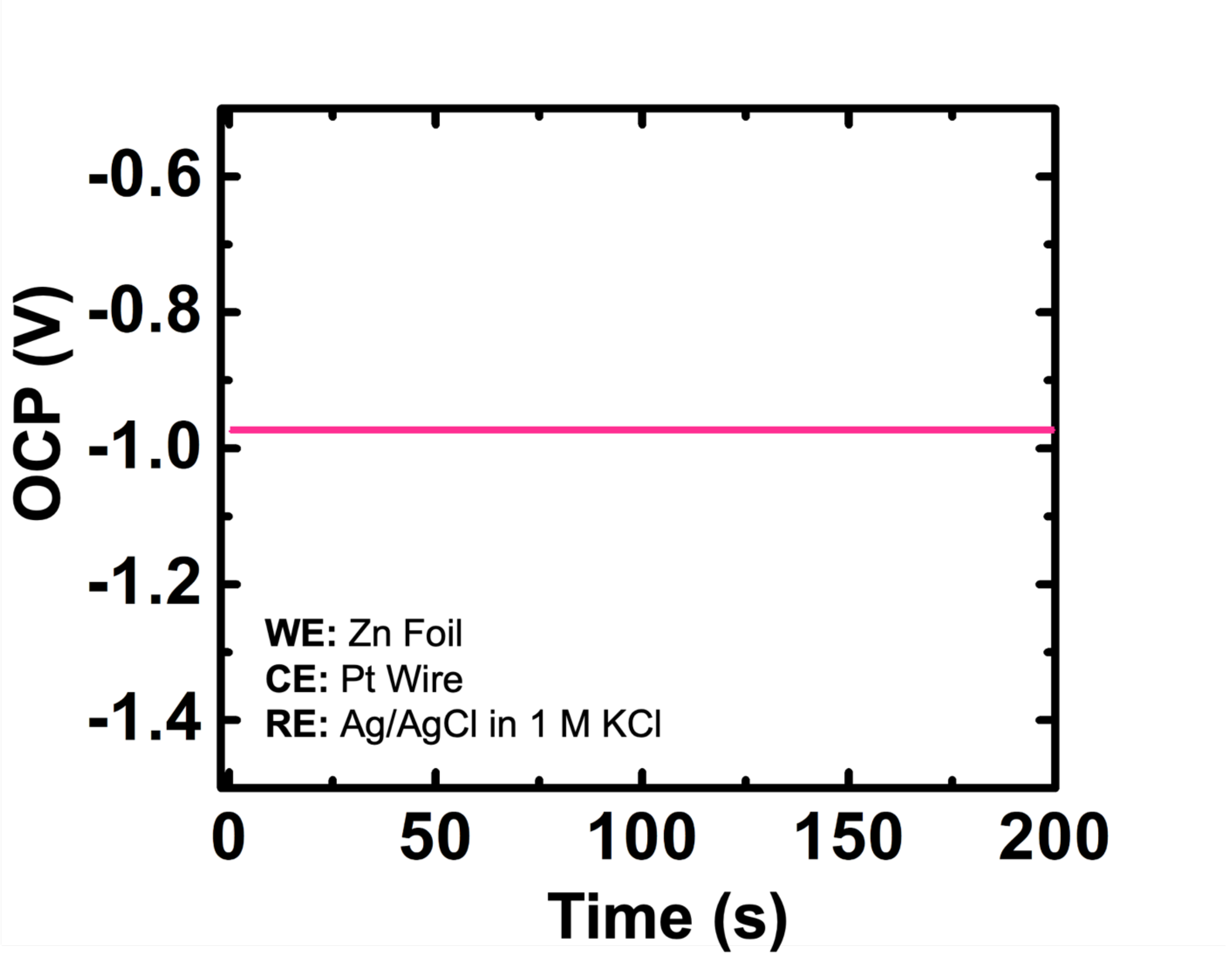

Fig S2. Open Circuit Potential (OCP) measurement using zinc foil as the working electrode, Pt wire as the counter electrode, and Ag/AgCl in 1 M KCl as the reference electrode.

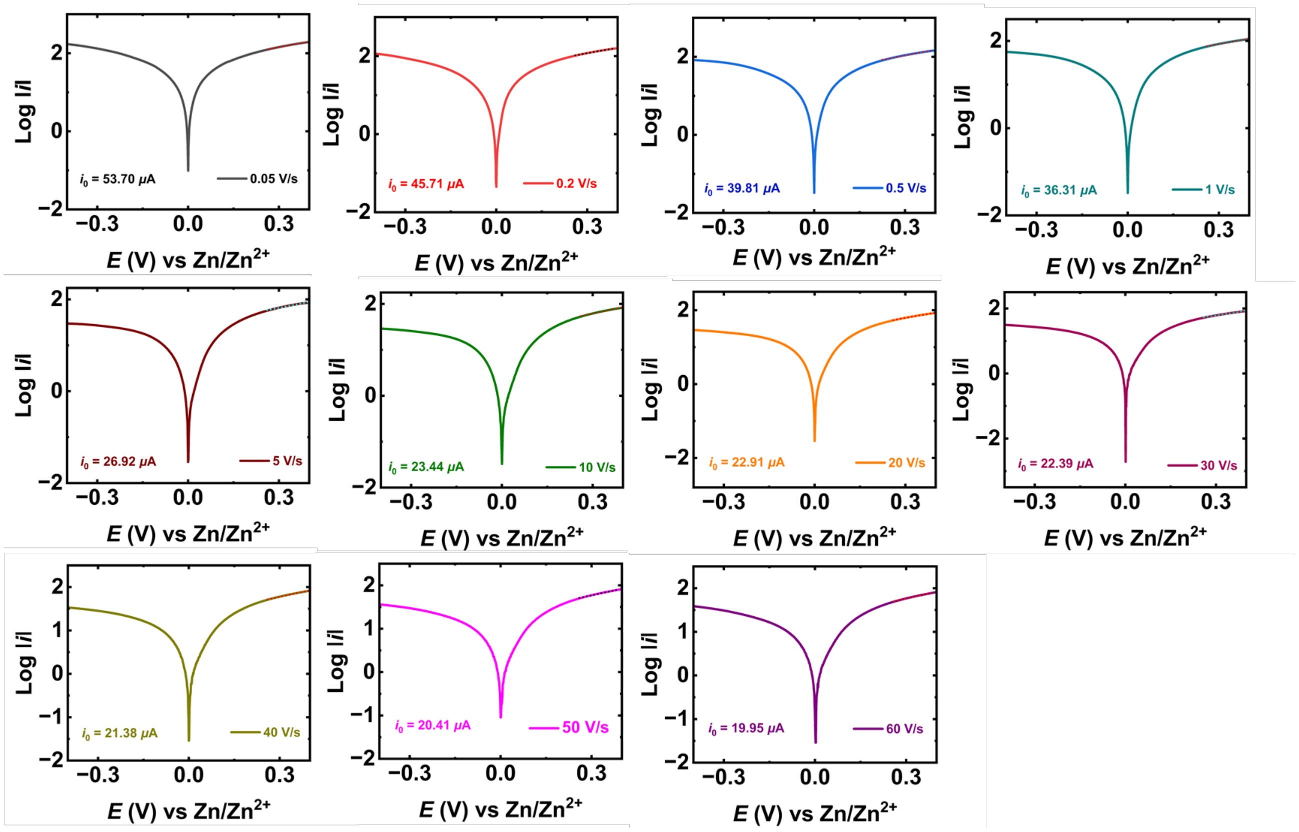

Fig S3. Tafel plots for all the scan rates using the methodology detailed in the main file.

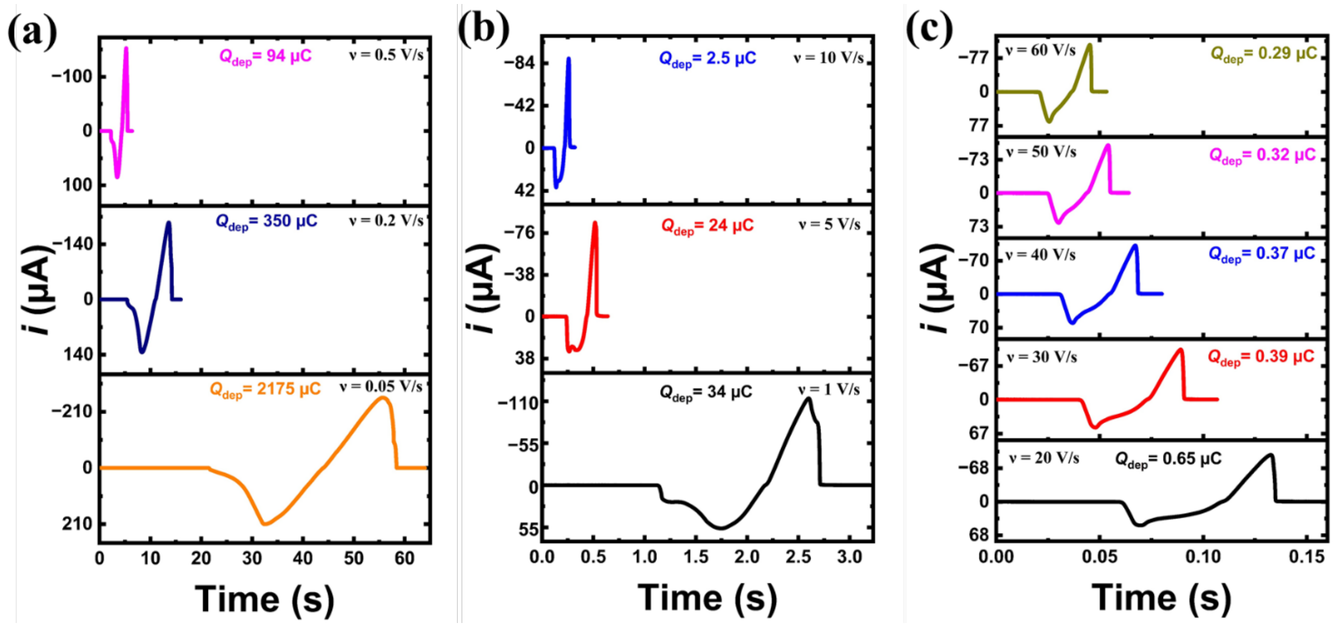

Fig. S4 Current vs time plot of Zn electrodeposition on 12.5 µm rad. W ultramicroelectrode at scan rates (a) 0.05 Vs^{-1}, 0.2 Vs^{-1}, 0.5 Vs^{-1}, (b) 1 Vs^{-1}, 5 Vs^{-1}, 10 Vs^{-1}, (c) 20 Vs^{-1}, 30 Vs^{-1}, 40 Vs^{-1}, 50 Vs^{-1}, 60 Vs^{-1}

Table S1. Peak current (i_p), deposited charge (Q_{dep}), exchange current (i_0), Tafel slope (m), and CE (%) as a function of scan rate (v).

$v\ (V/s)$	$i_p\ (\mu A)$	$Q_{dep}\ (\mu C)$	$i_0 (\mu A)$	$m\ (\mu A/V)$	CE (%)
0.05	262	2175	54	1.42	99.5
0.2	194	350	46	1.39	99.8
0.5	151	94	40	1.47	99.8
1	113	34	36	1.24	99.7
5	84	24	26	1.28	99.8
10	89	2.5	23	1.40	94.3
20	95	0.65	23	1.44	99.0
30	99	0.39	22	1.46	99.3
40	101	0.37	21	1.49	99.2
50	104	0.32	20	1.53	97.4
60	107	0.29	19	1.56	98.1

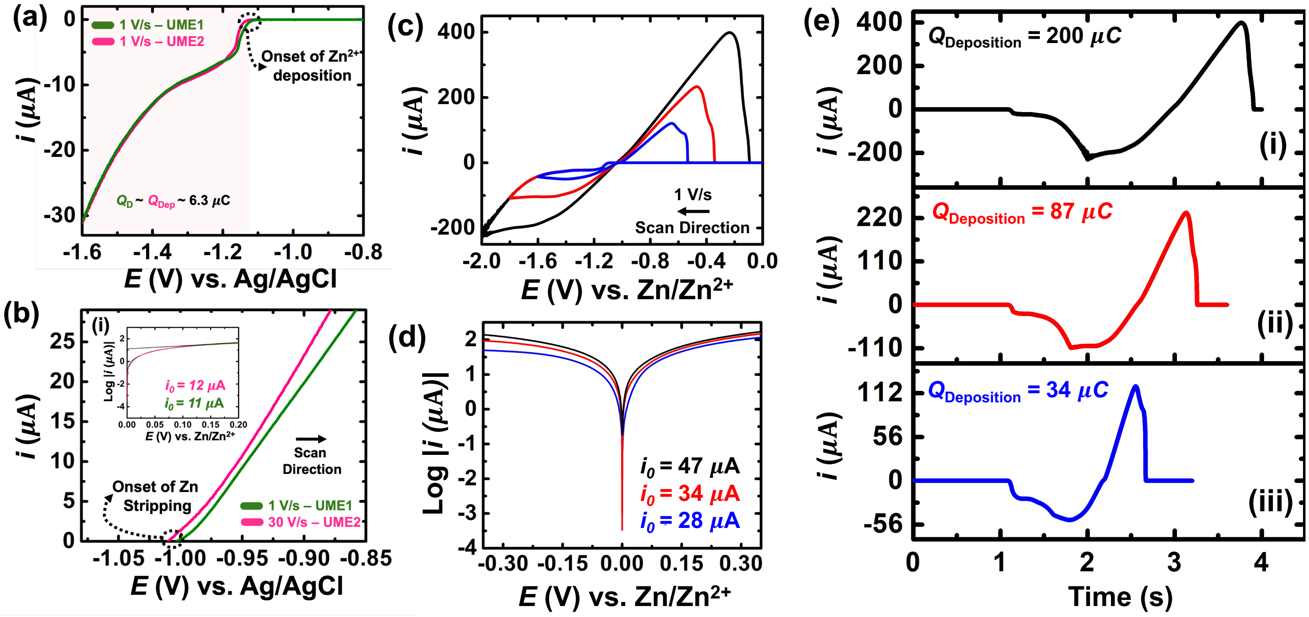

Fig. S5 (a) Linear sweep voltammogram recorded at a scan rate of 1 V/s to deposit a similar amount of zinc on two different 25 um tungsten electrodes. (b) Linear sweep voltammogram from the crossover potential to a more positive potential to show stripping of zinc on the two electrodes. (c) Cyclic voltammograms recorded at a scan rate of 1 V/s with variations in the potential at which the voltammetric sweep is reversed (-1.6 V, -1.8 V, and -2 V vs. Ag/AgCl). (d) Tafel plots corresponding to the kinetic regimes associated with voltammograms presented in (c). (d) Deposited charge ($Q_{\text{Deposition}}$) for the three cases shown in (c).

References

1. Bard, A. J.; Faulkner, L. R. *Electrochemical Methods : Fundamentals and Applications*. *John Wiley and Sons*, 2000, ISBN: 978-0-471-04372-0
